# 3-Methyl­sulfanyl-5-phenyl-1,2,4-triazine

**DOI:** 10.1107/S1600536814011830

**Published:** 2014-05-31

**Authors:** Salha Hamri, Abderrafia Hafid, Mostafa Khouili, Lahcen El Ammari, El Mostafa Ketatni

**Affiliations:** a Laboratoire de Chimie Organique et Analytique, Université Sultan Moulay Slimane, Faculté des Sciences et Techniques, BP 523, 23000 Béni-Mellal, Morocco; b Laboratoire de Chimie du Solide Appliquée, Faculté des Sciences, Université Mohammed V-Agdal, Avenue Ibn Battouta, BP 1014, Rabat, Morocco; c Laboratoire de Spectrochimie Applique et Environnement, Université Sultan Moulay Slimane, Faculté des Sciences et Techniques, BP 523, 23000 Béni-Mellal, Morocco

## Abstract

In the mol­ecule of the title compound, C_10_H_9_N_3_S, the dihedral angle between the triazine and phenyl rings is 11.77 (7)°. In the crystal, mol­ecules are linked by π–π stacking inter­actions [centroid–centroid distances = 3.7359 (3) and 3.7944 (4) Å], forming layers parallel to the *bc* plane.

## Related literature   

For the biological activity of sulfonamides, see: Abd el-Samii (1992 ▶); Kidwai *et al.* (1998 ▶); Holla *et al.* (2001 ▶); Abdel-Rahman *et al.* (1999 ▶); Hay *et al.* (2004 ▶); Sztanke *et al.* (2005 ▶). For the structure of a similar compound, see: Wen *et al.* (2006 ▶).
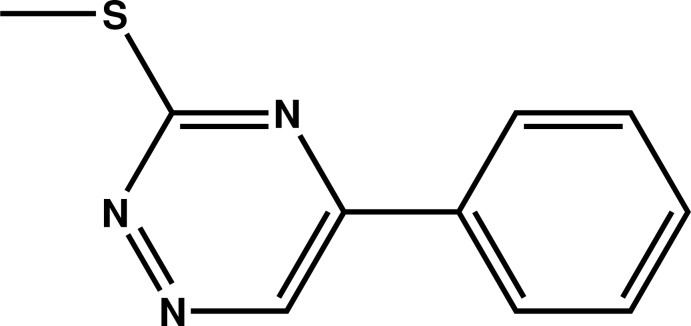


## Experimental   

### 

#### Crystal data  

C_10_H_9_N_3_S
*M* *_r_* = 203.26
Monoclinic, 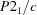

*a* = 7.7513 (3) Å
*b* = 12.9191 (5) Å
*c* = 9.8262 (3) Å
β = 94.584 (2)°
*V* = 980.85 (6) Å^3^
*Z* = 4
Mo *K*α radiation
μ = 0.29 mm^−1^
*T* = 296 K
0.41 × 0.35 × 0.29 mm

#### Data collection  

Bruker X8 APEX diffractometer
Absorption correction: multi-scan (*SADABS*; Bruker, 2009 ▶) *T*_min_ = 0.693, *T*_max_ = 0.747
15588 measured reflections
2859 independent reflections
2544 reflections with *I* > 2σ(*I*)
*R*_int_ = 0.023

#### Refinement  

*R*[*F* ^2^ > 2σ(*F* ^2^)] = 0.037
*wR*(*F* ^2^) = 0.110
*S* = 1.10
2859 reflections
127 parameters
H-atom parameters constrained
Δρ_max_ = 0.32 e Å^−3^
Δρ_min_ = −0.24 e Å^−3^

### 

Data collection: *APEX2* (Bruker, 2009 ▶); cell refinement: *SAINT* (Bruker, 2009 ▶); data reduction: *SAINT*; program(s) used to solve structure: *SHELXS97* (Sheldrick, 2008 ▶); program(s) used to refine structure: *SHELXL97* (Sheldrick, 2008 ▶); molecular graphics: *ORTEP-3 for Windows* (Farrugia, 2012 ▶); software used to prepare material for publication: *PLATON* (Spek, 2009 ▶) and *publCIF* (Westrip, 2010 ▶).

## Supplementary Material

Crystal structure: contains datablock(s) I. DOI: 10.1107/S1600536814011830/rz5126sup1.cif

Structure factors: contains datablock(s) I. DOI: 10.1107/S1600536814011830/rz5126Isup2.hkl

Supporting information file. DOI: 10.1107/S1600536814011830/rz5126Isup3.cml

CCDC reference: 1004529

Additional supporting information:  crystallographic information; 3D view; checkCIF report

## Figures and Tables

**Fig. 1.**
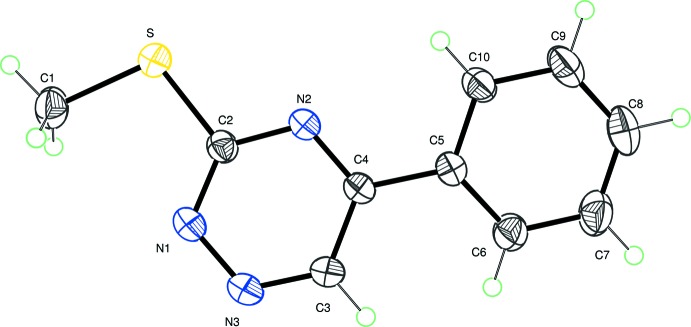
The molecular structure of the title compound with displacement ellipsoids drawn at the 50% probability level. H atoms are represented as small circles.

## supplementary crystallographic information

### 1. Comment

1,2,4-Triazine derivatives have been widely studied in terms of their synthetic methodologies and reactivity since some of these compounds were reported to have promising biological activities, including antimicrobial, inflammatory, analgesic, antiviral and anthelminitic activities (Abd el-Samii, 1992; Kidwai *et al.*, 1998; Holla *et al.*, 2001; Abdel-Rahman *et al.*, 1999; Hay *et al.*, 2004; Sztanke *et al.*, 2005). We synthesized the title compound and describe its structure here.

The molecular structure of 3-methylsulfanyl-5-phenyl-1,2,4-triazine is shown in Fig. 1. The triazine ring (N1–N3/C2–C4) forms a dihedral angle of 11.77 (7)° with the phenyl ring. Bond lengths and angles are compatible with those found in a related compound (Wen *et al.*, 2006). The cohesion of the crystal structure is ensured by π···π stacking interaction between triazine and phenyl rings [intercentroid distance = 3.7944 (4) Å] and between centrosymmetrically related triazine rings [intercentroid distance = 3.7359 (3) Å], forming molecular layers parallel to the *bc* plane.

### 2. Experimental

To a solution of thiosemicarbazide (50 g, 0.55 mol) in absolute ethanol (500 ml) was added iodomethane (34.1 ml, 0.55 mol). The reaction mixture was stirred at reflux for 3 h then cooled overnight in the refrigerator. The crystals formed were filtered on a Buchner funnel, washed with ethanol and dried *in vacuo* to give the *S*-methylthiosemicarbazide iodohydrate beige powder (123 g). An aqueous solution (200 ml) of *S*-methylthiosemicarbazide iodohydrate (20 g, 85.8 mmol) was added to an aqueous solution (120 ml) of phenyl glyoxal (15.65 g, 103 mmol) and sodium bicarbonate (10 g, 94.4 mmol) at 5°C. The temperature was maintained at 5°C for 6 h then the reaction medium was extracted with dichloromethane. The organic phases are combined, dried over magnesium sulfate, filtered and evaporated under reduced pressure. The crude material was purified by column on silica (hexane/EtOAc 9:1 *v*/*v*) as eluent. The title compound (13.95 g) was obtained as yellow crystals (79% yield) on slow evaporation of the solvent.

### 3. Refinement

H atoms were located in a difference Fourier map and treated as riding, with C–H = 0.93–0.96 Å and *U*_iso_(H) = 1.2 *U*_eq_(C) or 1.5 *U*_eq_(C) for methyl H atoms. One outlier (-1 7 1) was omitted in the last cycles of refinement.

### Crystal data

**Table:**

C_10_H_9_N_3_S	*F*(000) = 424
*M**_r_* = 203.26	*D*_x_ = 1.376 Mg m^−^^3^
Monoclinic, *P*2_1_/*c*	Mo *K*α radiation, λ = 0.71073 Å
Hall symbol: -P 2ybc	Cell parameters from 2859 reflections
*a* = 7.7513 (3) Å	θ = 2.6–30.0°
*b* = 12.9191 (5) Å	µ = 0.29 mm^−^^1^
*c* = 9.8262 (3) Å	*T* = 296 K
β = 94.584 (2)°	Block, yellow
*V* = 980.85 (6) Å^3^	0.41 × 0.35 × 0.29 mm
*Z* = 4	

### Data collection

**Table:**

Bruker X8 APEX diffractometer	2859 independent reflections
Radiation source: fine-focus sealed tube	2544 reflections with *I* > 2σ(*I*)
Graphite monochromator	*R*_int_ = 0.023
φ and ω scans	θ_max_ = 30.0°, θ_min_ = 2.6°
Absorption correction: multi-scan (*SADABS*; Bruker, 2009)	*h* = −10→10
*T*_min_ = 0.693, *T*_max_ = 0.747	*k* = −18→16
15588 measured reflections	*l* = −11→13

### Refinement

**Table:**

Refinement on *F*^2^	Primary atom site location: structure-invariant direct methods
Least-squares matrix: full	Secondary atom site location: difference Fourier map
*R*[*F*^2^ > 2σ(*F*^2^)] = 0.037	Hydrogen site location: inferred from neighbouring sites
*wR*(*F*^2^) = 0.110	H-atom parameters constrained
*S* = 1.10	*w* = 1/[σ^2^(*F*_o_^2^) + (0.0567*P*)^2^ + 0.210*P*] where *P* = (*F*_o_^2^ + 2*F*_c_^2^)/3
2859 reflections	(Δ/σ)_max_ = 0.001
127 parameters	Δρ_max_ = 0.32 e Å^−^^3^
0 restraints	Δρ_min_ = −0.24 e Å^−^^3^

### Special details

**Table:**

Geometry. All e.s.d.'s (except the e.s.d. in the dihedral angle between two l.s. planes) are estimated using the full covariance matrix. The cell e.s.d.'s are taken into account individually in the estimation of e.s.d.'s in distances, angles and torsion angles; correlations between e.s.d.'s in cell parameters are only used when they are defined by crystal symmetry. An approximate (isotropic) treatment of cell e.s.d.'s is used for estimating e.s.d.'s involving l.s. planes.
Refinement. Refinement of *F*^2^ against all reflections. The weighted *R*-factor *wR* and goodness of fit *S* are based on *F*^2^, conventional *R*-factors *R* are based on *F*, with *F* set to zero for negative *F*^2^. The threshold expression of *F*^2^ > σ(*F*^2^) is used only for calculating *R*-factors(gt) *etc*. and is not relevant to the choice of reflections for refinement. *R*-factors based on *F*^2^ are statistically about twice as large as those based on *F*, and *R*- factors based on all data will be even larger.

### Fractional atomic coordinates and isotropic or equivalent isotropic displacement parameters (Å^2^)

**Table:**

	*x*	*y*	*z*	*U*_iso_*/*U*_eq_	
S	0.23977 (4)	0.83039 (3)	0.60310 (3)	0.04391 (12)	
N1	0.55958 (14)	0.87884 (9)	0.70043 (10)	0.0411 (2)	
N2	0.49883 (12)	0.86358 (7)	0.45807 (9)	0.0321 (2)	
N3	0.72612 (15)	0.90131 (11)	0.68242 (11)	0.0492 (3)	
C1	0.2219 (2)	0.83401 (12)	0.78375 (15)	0.0478 (3)	
H1A	0.1053	0.8176	0.8025	0.072*	
H1B	0.2509	0.9020	0.8177	0.072*	
H1C	0.2997	0.7844	0.8278	0.072*	
C2	0.45673 (14)	0.86159 (8)	0.58822 (11)	0.0315 (2)	
C3	0.77637 (16)	0.90344 (12)	0.55773 (13)	0.0434 (3)	
H3	0.8915	0.9188	0.5461	0.052*	
C4	0.66358 (14)	0.88349 (8)	0.44103 (11)	0.0308 (2)	
C5	0.72027 (15)	0.88557 (8)	0.30105 (11)	0.0324 (2)	
C6	0.89529 (17)	0.88858 (11)	0.27791 (14)	0.0439 (3)	
H6	0.9785	0.8887	0.3515	0.053*	
C7	0.9461 (2)	0.89133 (13)	0.14577 (16)	0.0537 (4)	
H7	1.0631	0.8939	0.1311	0.064*	
C8	0.8234 (2)	0.89027 (12)	0.03593 (15)	0.0539 (4)	
H8	0.8576	0.8917	−0.0526	0.065*	
C9	0.6501 (2)	0.88713 (12)	0.05779 (13)	0.0499 (3)	
H9	0.5677	0.8863	−0.0164	0.060*	
C10	0.59717 (17)	0.88521 (10)	0.18942 (12)	0.0403 (3)	
H10	0.4799	0.8837	0.2032	0.048*	

### Atomic displacement parameters (Å^2^)

**Table:**

	*U*^11^	*U*^22^	*U*^33^	*U*^12^	*U*^13^	*U*^23^
S	0.03440 (17)	0.0619 (2)	0.03562 (17)	−0.00433 (12)	0.00418 (12)	0.00196 (13)
N1	0.0413 (5)	0.0550 (6)	0.0266 (4)	−0.0031 (4)	0.0003 (4)	0.0005 (4)
N2	0.0327 (4)	0.0367 (5)	0.0264 (4)	−0.0001 (3)	0.0000 (3)	0.0010 (3)
N3	0.0410 (6)	0.0750 (8)	0.0305 (5)	−0.0083 (5)	−0.0042 (4)	−0.0019 (5)
C1	0.0498 (7)	0.0555 (8)	0.0397 (7)	−0.0018 (6)	0.0137 (5)	0.0043 (5)
C2	0.0339 (5)	0.0325 (5)	0.0280 (5)	0.0015 (4)	0.0020 (4)	0.0023 (4)
C3	0.0328 (5)	0.0640 (8)	0.0324 (6)	−0.0063 (5)	−0.0023 (4)	−0.0007 (5)
C4	0.0330 (5)	0.0318 (5)	0.0271 (5)	0.0006 (4)	−0.0003 (4)	0.0008 (4)
C5	0.0374 (5)	0.0321 (5)	0.0280 (5)	0.0004 (4)	0.0033 (4)	−0.0002 (4)
C6	0.0389 (6)	0.0537 (7)	0.0396 (6)	0.0064 (5)	0.0060 (5)	0.0013 (5)
C7	0.0509 (8)	0.0642 (9)	0.0485 (8)	0.0080 (7)	0.0197 (6)	0.0016 (7)
C8	0.0753 (10)	0.0539 (8)	0.0350 (6)	0.0041 (7)	0.0192 (6)	−0.0014 (6)
C9	0.0675 (9)	0.0546 (8)	0.0271 (5)	−0.0046 (6)	−0.0002 (5)	−0.0023 (5)
C10	0.0443 (6)	0.0467 (6)	0.0294 (5)	−0.0060 (5)	−0.0006 (4)	−0.0012 (5)

### Geometric parameters (Å, º)

**Table:**

S—C2	1.7469 (11)	C4—C5	1.4773 (15)
S—C1	1.7921 (14)	C5—C6	1.3939 (17)
N1—C2	1.3268 (15)	C5—C10	1.3951 (16)
N1—N3	1.3486 (16)	C6—C7	1.3872 (18)
N2—C4	1.3263 (14)	C6—H6	0.9300
N2—C2	1.3451 (14)	C7—C8	1.381 (2)
N3—C3	1.3153 (17)	C7—H7	0.9300
C1—H1A	0.9600	C8—C9	1.378 (2)
C1—H1B	0.9600	C8—H8	0.9300
C1—H1C	0.9600	C9—C10	1.3880 (17)
C3—C4	1.4091 (15)	C9—H9	0.9300
C3—H3	0.9300	C10—H10	0.9300
			
C2—S—C1	103.19 (6)	C6—C5—C10	119.03 (11)
C2—N1—N3	116.42 (10)	C6—C5—C4	121.21 (10)
C4—N2—C2	115.65 (9)	C10—C5—C4	119.76 (11)
C3—N3—N1	118.99 (10)	C7—C6—C5	120.43 (13)
S—C1—H1A	109.5	C7—C6—H6	119.8
S—C1—H1B	109.5	C5—C6—H6	119.8
H1A—C1—H1B	109.5	C8—C7—C6	120.11 (14)
S—C1—H1C	109.5	C8—C7—H7	119.9
H1A—C1—H1C	109.5	C6—C7—H7	119.9
H1B—C1—H1C	109.5	C9—C8—C7	119.87 (12)
N1—C2—N2	127.71 (10)	C9—C8—H8	120.1
N1—C2—S	119.19 (9)	C7—C8—H8	120.1
N2—C2—S	113.10 (8)	C8—C9—C10	120.68 (13)
N3—C3—C4	122.93 (11)	C8—C9—H9	119.7
N3—C3—H3	118.5	C10—C9—H9	119.7
C4—C3—H3	118.5	C9—C10—C5	119.88 (13)
N2—C4—C3	118.28 (10)	C9—C10—H10	120.1
N2—C4—C5	118.82 (10)	C5—C10—H10	120.1
C3—C4—C5	122.89 (10)		
